# The Smc5/6 complex and the difficulties cutting the ties of twin sisters

**DOI:** 10.18632/aging.100295

**Published:** 2011-03-10

**Authors:** Jean-François Noël, Raymund J. Wellinger

**Affiliations:** Department of Microbiology and Infectious Diseases, Faculty of Medicine, Université de Sherbrooke, 3001 12e Ave N, Sherbrooke, Que, J1H 5N4, Canada

The fundamental process of chromosome segregation requires chromatin compaction and establishment of physical connections between sister chromatids. At the heart of these chromosome reorganizations reside three complexes formed by Structural Maintenance of Chromosome (SMC) proteins. Condensin (Smc2/4) is in charge of chromosome compaction [1] while cohesin (Smc1/3) holds together sister chromatids after replication [2]. Together with the third complex, Smc5/6, these are associated with DNA repair. However, linkages between chromatids are not only mediated by cohesin. For example, intertwinings between sister chromatids are produced by the DNA replication process itself. Furthermore, since replication forks connect sister chromatids, incomplete DNA replication would impair chromosome segregation. Finally, the process of DNA repair by homologous recombination (HR) generates intermediates linking chromatids. Failure to remove these linkages in a timely fashion at mitosis can induce massive genome instability and chromosome rearrangements which are hallmarks of cancer cells [3].

Although the link between Smc5/6 and DNA repair was known for some time, details of Smc5/6 functions are just beginning to be uncovered. For example, Smc5/6 promotes DNA double-strand break repair by sister-chromatid HR [4]. A number of studies also associate Smc5/6 with repair of stalled/collapsed replication forks since replication through damaged templates caused an accumulation of X-shaped DNA structures in smc5/6 mutants [5, 6, 7]. It was initially thought that Smc5/6 had a non-essential function in DNA repair and an essential function in chromosome segregation since affecting Smc5/6 hinders segregation of repetitive chromosomal regions like the rDNA locus and telomeres [8].

However, the distinction between functions in DNA repair versus chromosome segregation of Smc5/6 is unclear. It was proposed that Smc5/6 resolves particular HR intermediates generated in an attempt to repair stalled or defective replication forks [5, 6, 9]. Failure to resolve these chromatid-linking structures would lead to chromosome breakage at cell division. Accordingly, inactivation of Smc5/6 results in DNA damage checkpoint activation only in the following cell cycle [6, 8], which is consistent with damages occurring during mitosis. At least three points are left unexplained in this model. First, to allow the cell to go on with mitosis, these DNA linkages must be structures not recognized by any pre-mitotic checkpoints. Alternatively, these structures would only be undetectable when arising at specific genomic locations. What those specific structures are, however, remains nebulous. Second, HR abolition only partially rescues smc5/6 mutant phenotypes, suggesting a poorly understood HR-independent function. Third, the model does not explain the function of the complex in unchallenged cells. Part of the answers to these nagging questions could come from the proposition that failure to disjoin rDNA in smc5/6 mutants is caused by cell division in presence of normal ongoing replication forks at the rDNA locus [10]. However, Smc5/6 functions are not limited to the rDNA array and it was proposed that the complex also functions in timely replication and resolution of telomeres [8, 11].

The implication of Smc5/6 in telomere separation is intriguing as Smc5/6 appears to have intricate connections to telomere biology. For example, Smc5/6 is enriched at budding yeast telomeres [8] and mutants show slight defects in telomere maintenance [12]. Moreover, Smc5/6 functions in the ALT (Alternative Lengthening of Telomeres) telomere maintenance pathway, a HR-dependant telomerase-independent pathway active in 10-15% of cancers [13]. Smc5/6 depletion in ALT cells inhibits telomere recombination, causing telomere shortening and cell senescence [14]. Finally, Smc5/6 appears to counteract accumulation of HR structures at telomeres in senescing telomerase negative yeast cells [5]. Telomeres are particularly susceptible to defects in replication fork progression. Their heterochromatic nature and the interference of DNA-protein complexes and higher-order DNA secondary structures can induce frequent replication pausing. Furthermore, a fork stalled in telomeric repeats cannot be rescued by a converging fork since telomeres are replicated in a unidirectional way. At least in budding yeast, telomeres are amongst the last areas in the genome to be replicated, increasing the possibility of escaping checkpoint control and enter mitosis with unreplicated DNA.

In yeast smc5/6 mutants, sister telomeres still connected at mitosis by a combination of ongoing replication forks and unresolved HR intermediates could break upon cell division. However, such occasional breakage occurring inside telomeric repeats can still be repaired by telomerase elongation in the following cell cycle. In telomerase negative yeast cells, breaks in terminal repeat DNA will cause abrupt loss of telomeric DNA and this shortening will eventually result in cell division arrest after fewer generations than normally observed [5, 11]. This cell division arrest occurs even more rapidly in HR-defective cells, consistent with a HR-independent contribution to telomere stability by Smc5/6 [11]. Accordingly, a recent study implicates Smc5/6 in the resolution of replication-induced topological stress in undamaged yeast cells [15]. Finally, and as predicted by these ideas,sequenced telomeres from telomerase-positive smc5/6 mutant yeast cells revealed a higher proportion of divergent distal telomeric repeat sequences, suggesting a higher frequency of telomere breakage events [11]. A recent analysis of smc5/6 mutants also revealed enhanced frequency of chromosome breakage during an otherwise unperturbed mitotic cell division [16].

In conclusion, at least at telomeres, Smc5/6 participates in efficient replication and repair to avoid stochastic sequence losses during cell division. In order to do so, the complex is involved in a HR-dependent repair pathway as well as a HR-independent pathway, possibly implicating resolution of topological intertwinings. However, there is potential for even more implications for Smc5/6 in manipulating chromosome architecture: the Mms21 SUMO-ligase subunit of the Smc5/6 complex was recently shown to be involved in sumoylation of all three SMC complexes [12, 17]. Therefore, this third complex, although a late starter in terms of discovered details of its functions, could eventually outshine the other two in its complexity and scope of involvement to assure genome stability.
